# Prenatal Diagnostic Value of Chromosomal Microarray in Fetuses with Nuchal Translucency Greater than 2.5 mm

**DOI:** 10.1155/2019/6504159

**Published:** 2019-10-03

**Authors:** Zhu Zhang, Ting Hu, Jiamin Wang, Qinqin Li, He Wang, Shanling Liu

**Affiliations:** ^1^Prenatal Diagnosis Center, West China Second University Hospital, Sichuan University, Chengdu, Sichuan, China; ^2^Key Laboratory of Birth Defects and Related Diseases of Women and Children (Sichuan University), Ministry of Education, Chengdu, China

## Abstract

**Objective:**

To assess the clinical value of prenatal diagnosis using quantitative fluorescent polymerase chain reaction (QF-PCR) and chromosomal microarray analysis (CMA) for the examination of genomic imbalances in prenatal amniotic fluid samples from fetuses with a nuchal translucency (NT) greater than or equal to 2.5 mm.

**Materials and Methods:**

A total of 494 amniotic fluid samples and 5 chorionic villus samples were included in this study, with a fetal NT ≥ 2.5 mm at 11–13^+6^ weeks of gestation from November 2015 to December 2018. All cases were examined with QF-PCR, and those with normal QF-PCR results were then analyzed by CMA.

**Results:**

Of the 499 cases, common aneuploidies were detected by QF-PCR in 61 (12.2%) cases. One case of triploidy, one case of trisomy 21 mosaicism, and two cases of X/XX mosaicism were further confirmed by fluorescence in situ hybridization (FISH). Among the 434 cases with normal QF-PCR results, microarray detected additional pathogenic copy number variants (CNVs) in 4.8% (21/434) of cases. Six cases would have been expected to be detectable by conventional karyotyping because of large deletions/duplications (>10 Mb), leaving fifteen (3.5%, 15/428) cases with pathogenic CNVs only detectable by CMA. Pathogenic CNVs, especially those <10 Mb, were centralized in cases with an NT < 4.5 mm, including 5 pathogenic CNVs in cases with an NT of 2.5–3.5 mm and 7 pathogenic CNVs in cases with an NT of 3.5–4.5 mm.

**Conclusions:**

It is rational to use a diagnostic strategy in which CMA is preceded by a less-expensive, rapid method, namely, QF-PCR, to detect common aneuploidies. CMA allows for the detection of a number of pathogenic chromosomal aberrations in fetuses with an NT ≥ 2.5 mm.

## 1. Introduction

Nuchal translucency (NT) refers to the collection of fluid in the back of the fetal neck [1]. Measurement of nuchal translucency (NT) between 11 and 13^+6^ weeks above the 99^th^ centile (≥3.5 mm) is defined as increased NT. Increased NT thickness is associated with fetal structural defects, chromosomal abnormalities, and genetic disorders [2–7]. Some congenital malformations, which mainly involve congenital heart disease, diaphragmatic hernia, and orofacial clefts, are increased in cases with an NT ≥ 3.5 mm [8–10]. Common aneuploidies, including trisomies 21, 18, and 13 and monosomy X, are the major chromosomal abnormalities associated with increased NT. Genetic disorders have also been reported in association with enlarged NT. The most common conditions include Noonan syndrome, Smith–Lemli–Opitz syndrome, and congenital adrenal hyperplasia [7, 11, 12].

CMA detects imbalances in the DNA copy number, which are referred to as copy number variants (CNVs) [13]. Chromosomal microarray analysis (CMA) shows advantages over conventional karyotyping not only in postnatal diagnosis but also in prenatal diagnosis. Especially in cases with structural anomalies found during prenatal imaging examinations, CMA can detect another 5.6% pathogenic copy number variants (CNVs) in isolated defects and 9.1% pathogenic CNVs in multiple defects [14]. Recent studies have concluded different detection rates for pathogenic CNVs in cases with increased NT (0–15%) [15–17]. Some microdeletion/microduplication syndromes, including 22q11.2 deletion syndrome, have been found to be associated with enlarged NT thickness [18, 19].

Though in most studies, increased NT has been defined as measurement greater than or equal to 3.5 mm, we found that some pathogenic CNVs could also be detected in cases with an NT equal to or greater than 2.5 mm but less than 3.5 mm in our routine work. Therefore, the objective of this study was to assess the performance of a prenatal diagnostic strategy using combined QF-PCR and CMA for fetuses with an NT ≥ 2.5 mm.

## 2. Materials and Methods

This investigation was a retrospective study in which a total of 499 pregnancies with an NT ≥ 2.5 mm were enrolled at the Prenatal Diagnosis Center of West China Second University Hospital from November 2015 to December 2018. NT was assessed between 11 and 13^+6^ weeks (fetal crown-rump length ranging between 45 and 84 mm) according to the standards of the Fetal Medicine Foundation (FMF) by FMF-certified sonographers. All women received comprehensive prenatal counseling, including the possible outcome of the fetuses and the indications, accuracy, limitations, and risk of amniocentesis, QF-PCR, and CMA. Signed informed consent forms were obtained from all participants.

The initial 2 ml of amniotic fluid (AF) was abandoned to avoid maternal cell contamination. If maternal cell contamination (MCC) was suspected, DNA was extracted from cultured AF. If MCC was excluded, DNA was extracted immediately from the uncultured AF using the QIAamp® DNA Blood Mini Kit (QIAGEN GmbH, Hilden, Germany). All samples were subjected to QF-PCR detection using a trisomy 21, 18, and 13 and sex chromosome polyploidy detection kit (fluorescence PCR-capillary electrophoresis) (DAAN GENE, Guangzhou, China), following the manufacturer's protocol.

QF-PCR can detect the number of abnormalities of chromosomes 13, 18, 21, X, and Y. The PCR fragments were separated by capillary electrophoresis (3500, Life Technologies, CA, USA), and data were analyzed using GeneMapper® (version 4.1, Applied Biosystems, Waltham, Massachusetts, USA). Abnormal QF-PCR results were validated using fluorescence in situ hybridization (FISH).

If the QF-PCR result was normal, CMA would follow. All samples were screened using a CytoScan 750K array (Affymetrix, Inc., Santa Clara, CA, USA). The sensitivity and specificity were universally acknowledged, and CNVs equal to or greater than 200 kb across the genome could be reliably detected. The experimental procedures were performed according to the manufacturer's standard protocols (Affymetrix, Inc., Santa Clara, CA, USA), specifically including digestion, ligation, PCR amplification, purification, segmentation, labeling, and hybridization. The results were analyzed, respectively, by two clinical geneticists using the Chromosome Analysis Suite (ChAS) software (Affymetrix, Inc., Santa Clara, CA, USA). The results were determined using in-house databases and publicly available CNV databases, including the Database of Chromosomal Imbalance and Phenotype in Humans Using Ensembl Resources (DECIPHER; http://decipher.sanger.ac.uk), GeneReviews®, Database of Genomic Variants (DGV; http://projects.tcag.ca/variation), Online Mendelian Inheritance in Man (OMIM; http://www.omim.org), and ClinGen (https://www.clinicalgenome.org/). Sometimes, public databases lag behind the latest literature; consequently, PubMed was also involved in the data analysis process. In our study, a conventional cytogenetic analysis was not performed in addition to CMA. CNVs with a size greater than 10 Mb were considered visible by karyotyping, while CNVs <10 Mb were classified as cryptic. We categorized CNVs as benign, pathogenic, or variants of uncertain significance (VOUS) according to the American College of Medical Genetics (ACMG) standards and guidelines for the interpretation and reporting of postnatal constitutional CNVs [20]. If VOUS were detected in the fetal sample, peripheral blood was collected from both parents, and the results were further analyzed to differentiate the CNVs that were likely benign, likely pathogenic, or true VOUS. Clinical geneticists in our prenatal diagnosis center offered counseling to women with array results.

### 2.1. Statistical Analysis

GraphPad Prism, version 4.03 (GraphPad Software Inc., CA, USA), was used for statistical analysis. We used the chi-square test to assess for significant differences in expected frequencies between two groups. *P* < 0.05 was considered statistically significant in a two-sided test.

## 3. Results

**Figure 1:**
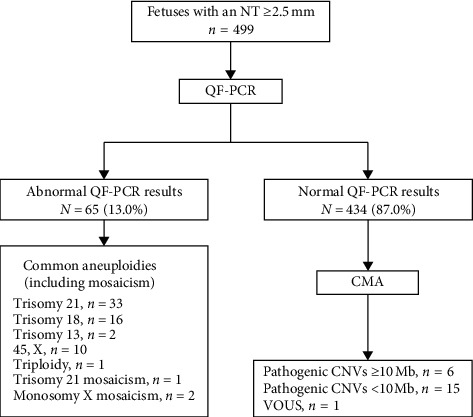
Flowchart of patient characteristics and chromosomal findings from quantitative fluorescent polymerase chain reaction (QF-PCR) and chromosomal microarray (CMA) of 499 amniotic fluid and chorionic villus samples from pregnancies with an nuchal translucency (NT) ≥2.5 mm. CNVs, copy number variants; VOUS, variants of uncertain significance.

Figure 1 shows the patient characteristics and chromosomal findings analyzed by QF-PCR and CMA (Figure 1). A total of 499 fetuses with an NT ≥ 2.5 mm were enrolled in this study. The median maternal age was 29.3 (range, 18–44) years, the median gestational age was 19^+3^ (range, 12^+4^–25^+5^) weeks, and the median fetal NT thickness was 3.5 (range, 2.5–9.7) mm. Nineteen cases had cystic hygroma. Of all cases, 13 were combined with other structural malformations, and the remaining 486 cases were with isolated increased NT (Table 1). The distribution of different types of CNVs according to the NT thickness is presented in Table 2. Of the 499 fetuses included, 61 (12.2%) were identified with aneuploidies involving chromosome 13, 18, 21, or X by QF-PCR analysis (Table 2). One case of trisomy 21 mosaicism, two cases of monosomy X mosaicism, and one case of triploidy were also detected. All abnormal QF-PCR results were verified by FISH. All women with affected pregnancies elected to terminate their pregnancies.

Of the remaining 434 cases, 21 (4.8%) had pathogenic CNVs detected by CMA. One (0.2%) case was of unclear clinical significance. The findings from CMA are summarized in Table 3. Fifteen (3.5%, 15/428) cases with imbalances detected by CMA had ≤10 Mb CNVs (range, 0.256–9.236 Mb), which would not have been detected by karyotype analysis. Table 1 shows the chromosomal findings in samples with other structural malformations. The number of aneuploidies including aneuploidy mosaicism in cases with other structural malformations (61.5%, 8/13) is much more than that in cases with isolated increased NT (11.7%, 57/486) (*P* < 0.001, OR 16.93, 95% CI 5.092–50.53). One case with an NT of 3.3 mm complicated by an obviously short femur was diagnosed with achondroplasia, and the CMA result was normal. However, neither pathogenic nor likely pathogenic CNVs were found in cases of increased NT with other anomalies.

In the cases with an NT ≥3.5 mm, 49 cases were diagnosed with chromosomal aneuploidies, one case was diagnosed with triploidy by QF-PCR (22.8%, 50/219), and another 15 (8.9%, 15/169) cases were found to have pathogenic CNVs by CMA. However, in the cases with an NT of 2.5–3.5 mm, 15 (5.4%, 15/280) cases were diagnosed with chromosomal aneuploidies (including one case of trisomy 21 mosaicism and two cases of monosomy X mosaicism) by QF-PCR and FISH, and another 6 (2.3%, 6/265) cases were found with pathogenic CNVs by CMA. Pathogenic CNVs, especially those <10 Mb, were centralized in cases with an NT < 4.5 mm, including 5 pathogenic CNVs in cases with an NT of 2.5–3.5 mm and 7 pathogenic CNVs in cases with an NT of 3.5–4.5 mm.

After genetic counseling, two patients with pregnancies exhibiting pathogenic CNVs continued their pregnancies, and the others terminated their pregnancies. One pregnancy exhibiting VOUS was continued, and a healthy infant was born.

## 4. Discussion

Increased NT was demonstrated to be an important screening method for chromosomal aneuploidies and fetal structural abnormalities. In this study, 86 cases of chromosomal abnormalities were diagnosed, including 61 (12.2%) cases of chromosomal aneuploidies, 21 (4.8%) cases of pathogenic CNVs, one case of triploidy, one case of trisomy 21 mosaicism, and two cases of X/XX mosaicism. However, different studies have reported different detection rates for chromosomal abnormalities. In Table 4, we compare our present study to recent publications. Lund et al. reported one of the highest diagnostic rates (28.8%, 38/132) of chromosomal aneuploidies and the highest detection rate (12.8%, 12/94) of pathogenic CNVs detected by CMA in fetuses with an NT ≥3.5 mm [17]. A French multicenter retrospective study reported one of the largest populations of increased NT and found that 16 (2.7%) pathogenic CNVs could be detected by CMA [16]. Pan et al. in China found that 5.7% of pathogenic CNVs could be detected by CMA [21]. However, Scott et al. found that only one pathogenic CNV was detected by CMA in 90 fetuses with an NT ≥ 3.5 mm [22]. In another study, Schou et al. found no additional benefit of microarray over karyotyping in 100 cases [15]. Huang et al. also reported that CMA could not find more pathogenic CNVs than karyotyping could [23]. Different detection rates among the studies might be caused by different CMA platforms used, the definition of “pathogenic CNV,” or different study sample sizes. Grande summarized 17 studies in the only one recently published meta-analysis that focused on the incremental yield of microarray over karyotyping in fetuses with increased NT [24]. This study reported 5% additional pathogenic CNVs in normal karyotype results, slightly higher than the results of our study (3.5%, 15/428).

In the existing literature, the threshold of increased NT was universally defined as equal to or greater than the 99^th^ centile (≥3.5 mm) [16, 17, 21, 25]. The rate of chromosomal aneuploidies and pathogenic CNVs both increased with NT thickness. Some other studies compared the chromosomal results in cases with an NT equal to or greater than the 95^th^ centile (≥3.0 mm) with those in cases with NT less than 3.0 mm [26]. The results of the study found that the karyotype was abnormal in 25 (69%) cases with an NT ≥ 3.0 mm. The authors also found that cases of trisomies 18 and 45 and XO occurred more often than those of trisomy 21 in this group. In seven cases with an NT < 3.0 mm, only one had an abnormal karyotype (47, +18). Another study concluded that the incidence of chromosomal abnormalities increased with NT thickness from approximately 7% for those with an NT between the 95^th^ centile for crown-rump length and 3.4 mm to 75% for those with an NT of 8.5 mm or more [25]. Whichever standard of increased NT the researchers chose, the studies consistently showed that, with an increase in NT thickness, the detection rates of chromosomal anomalies were accordingly improved.

Table 2 shows the distribution of chromosomal abnormalities of 499 cases with an NT ≥2.5 mm. In the cases with an NT of 2.5–3.5 mm, we found 15 (5.4%, 15/280) cases with chromosomal aneuploidies and 6 (2.3%, 6/265) cases with pathogenic CNVs. Interestingly, the detection rate of chromosomal aneuploidies in cases with an NT of 2.5–3.0 mm (5.5%, 8/146) was similar to that in cases with an NT of 3.0–3.5 mm (5.2%, 7/134). The detection rate of chromosomal aneuploidies increased with NT thickness (5.4% in cases with an NT of 2.5–3.5 mm vs. 47.1% in cases with an NT ≥ 6.5). However, pathogenic CNVs, especially those <10 Mb, were centralized in cases with an NT < 4.5 mm including 5 pathogenic CNVs in cases with an NT of 2.5–3.5 mm and 7 pathogenic CNVs in cases with an NT of 3.5–4.5 mm. Recently, Maya et al. reconsidered the cut-off value of increased NT thickness [27]. In their study, 770 fetuses had NT as a normal or an isolated abnormal finding according to the previous standard of NT thickening. Of these fetuses, 462 had an NT ≤ 2.9 mm, 170 had an NT of 3.0–3.4 mm, and 138 had an NT ≥ 3.5 mm. Pathogenic copy number variations were found in 1.7%, 7.1%, and 13.0% of the groups above, respectively. They concluded that CMA should be applied in fetuses with isolated, mildly increased NT (3.0–3.4 mm). However, the detection rate of pathogenic CNVs in cases with an NT of 2.5–3.0 mm was not mentioned in the study. In our study, 2 (3.0%, 4/134) pathogenic CNVs were diagnosed in patients with an NT equal to or greater than 2.5 mm and less than 3.0 mm. The range of NT thickness of Chinese fetuses needs to be reevaluated.

In our study, two cases of 22q11 deletion syndrome were identified by CMA. One case was diagnosed with an NT of 3.6 mm, and the other was diagnosed with an NT of 3.0 mm. Both cases were found to have congenital heart defects by ultrasound before pregnancy termination.

Notably, the pathogenic CNVs in our study include cases associated with a highly variable phenotype and an incomplete penetrance with clinically relevant CNVs. In Case 4, we detected a 16p11.2 microdeletion that is associated with developmental delay, intellectual disability, and/or autism spectrum disorder. Hanson et al. reported that the IQ average of those affected individuals was 82.7, representing a 26.8-point (1.8 SD) shift downward compared to the full-scale IQ average of 109.5 of familial controls [28]. In Case 5, in addition to trisomy 21 mosaicism, we detected a 16p13.11 recurrent microduplication, which is a neurocognitive disorder susceptibility locus associated with intellectual disability, attention-deficit hyperactivity disorder, and autism, but with variable penetrance [29–31]. Prenatal counseling of these cases must be conducted cautiously and necessitates a professional team including genetic counselors, clinical geneticists, and fetal medical experts. In the above two cases, the pregnancies were terminated. In Case 4, the 16p11.2 microdeletion was found to be de novo, and therefore, the risk of recurrence was low. The parents did not continue the pregnancy after detailed genetic counseling. In Case 5, the parents decided to terminate the pregnancy mainly because of the detection of trisomy 21 mosaicism.

The number of aneuploidies in cases with structural malformation was much more than that in cases with isolated increased NT. However, there were no pathogenic CNVs found in samples with other abnormalities. These might indicate that the structural anomalies caused by pathogenic CNVs were so small that could not be found in NT scanning.

The chromosomal result of one case was determined to be variants of unknown significance. The infant was born. Her father told us that her condition was satisfactory in the telephone follow-up when the baby was one year old.

## 5. Conclusion

This study showed that compared with karyotyping, CMA reveals a significantly additional number of clinically relevant pathogenic CNVs in 3.5% of fetuses with increased NT. However, in developing countries, QF-PCR combined with FISH could be used as a first-line method in cases with increased NT. If the rapid test results are normal, microarray analysis is effective as a prenatal testing regime for fetuses with a high NT without requiring conventional karyotyping. In addition, we must be very careful in cases with an NT between 2.5 and 3.5 mm because 5.4% of cases were detected to be chromosomal aneuploidies and 2.3% of cases were found to have pathogenic CNVs.

## Figures and Tables

**Table 1:** Chromosomal findings in samples with other structural malformations.

Case	MA (years)	NT (mm)	Other malformations	Chromosomal findings
1	33	3.6	VSD	Trisomy 21
2	29	3.3	Short and bending femur	Achondroplasia
3	33	3.6	Multiple malformations	N
4	35	2.7	Holoprosencephaly	Trisomy 13
5	27	3.5	Pedicle syndrome	N
6	28	2.7	CHD	Trisomy 21
7	37	4.8	Omphalocele	Trisomy 18
8	33	4.0	Omphalocele	Trisomy 18
9	33	3.2	Choroid plexus cysts	Trisomy 18
10	37	3.7	Holoprosencephaly	N
11	24	2.9	Situs inversus viscerum, CHD	Monosomy X mosaicism
12	36	7.2	Holoprosencephaly, omphalocele	Trisomy 18
13	33	3.5	Diaphragmatic hernia	N

VSD: ventricular septal defect; CHD: congenital heart disease; N: negative results.

**Table 2:** Distribution of chromosomal abnormalities detected by quantitative fluorescent polymerase chain reaction (QF-PCR) and chromosomal microarray analysis (CMA) of 499 amniotic fluid samples from pregnancies with a nuchal translucency (NT) ≥2.5 mm.

NT (mm)	*N* (%)	Aneuploidies and triploidy detected by QF-PCR							Pathogenic	CNVs	VOUS
		Trisomy 21	Trisomy 18	Trisomy 13	Monosomy X	Trisomy 21 mosaicism^b^	Monosomy X mosaicism^b^	Triploidy^b^	≥10 Mb	<10 Mb	
2.5 mm ≤ NT < 3.0 mm	134 (26.9)	2	1	1	0	1	2	0	1	3	1
3.0 mm ≤ NT < 3.5 mm	146 (29.3)	6	1	0	1	0	0	0	0	2	0
3.5 mm ≤ NT < 4.5 mm	140 (28.0)	14	6	0	3	0	0	0	0	7	0
4.5 mm ≤ NT < 5.5 mm	32 (6.4)	3	2	0	0	0	0	0	2	0	0
5.5 mm ≤ NT < 6.5 mm	13 (2.6)	4	0	0	1	0	0	1	1	1	0
6.5 mm^a^ ≤ NT	34 (6.8)	4	6	1	5	0	0	0	2	2	0

^a^Nineteen cases diagnosed with cystic hygroma were classified in this group. ^b^One case of trisomy 21 mosaicism, two cases of monosomy X mosaicism, and one case of triploidy were further confirmed by fluorescence in situ hybridization (FISH).

**Table 3:** Pathogenic copy number variants (CNVs) and variants of uncertain significance (VOUS) detected by chromosomal microarray analysis (CMA).

Case	MA (years)	GA at amniocentesis or CVS (weeks)	NT (mm) CRL (cm)	CMA results	Size (Mb)	Inheritance	Gene affected or syndromes	Categorization	Pregnancy outcome
1	23	18^+2^	5.1 6.8	arr8q23.1q24.21(108,615,421-129,519,596)x1	20.904	De novo	47 OMIM, 13 morbidity	Pathogenic (unique)	TOP
2	23	17^+5^	8.0 6.1	arr 18q22.3q23(71,975,414-78,013,728)x1	6.038	De novo	17 OMIM, 3 morbidity	Likely pathogenic (unique)	TOP
3	30	19^+2^	2.7 4.6	arr 17p13.3p13.2(525-3,613,691)x1	3.613	De novo	Miller–Dieker syndrome	Pathogenic (unique)	TOP
4	31	18^+1^	4.1 4.9	arr 16p11.2(29,428,531-30,190,029)x1	0.761	De novo	16p11.2 recurrent microdeletion	Pathogenic (known)	TOP
5	23	22^+4^	2.6 5.3	arr21q11.2q22.3(15,016,486-48,093,361)x2.63 arr[hg19] 16p13.11(14,929,070-16,289,059)x3	33.1 1.360	De novo	Trisomy 21 mosaicism 16p13.11 recurrent microduplication	Likely pathogenic (known)	TOP
6	33	21^+5^	3.3 7.0	arr Yq11.221q11.23(19,563,599-28,799,654)x0	9.236	De novo	AZFb + AZFc	Pathogenic (unique)	TOP
7	26	18	3.7 6.8	arr 17q12(34,822,465-36,307,773)x1	1.485	De novo	Renal cysts and diabetes syndrome (RCAD)	Pathogenic (known)	TOP
8	25	20	5.1 6.8	arr 4q28.1q34.3(127,146,008-180,134,001)x3	52.988	De novo	112 OMIM, 29 morbidity	Pathogenic (unique)	TOP
9	36	19^+2^	6.5 6.6	arr 21q21.3(27,328,142-27,584,525)x3	0.256	Unknown	Early-onset Alzheimer's disease with cerebral amyloid angiopathy	Pathogenic (unique)	Live birth
10	27	20^+1^	3.6 5.3	arr 22q11.21(18,631,364-21,800,471)x1	3.169	De novo	22q11 deletion syndrome	Pathogenic (known)	TOP
11	28	18^+3^	4.0 4.6	arr 1q21.1q21.2(144,494,997-148,661,621)x1	4.167	De novo	1q21.1 recurrent microdeletion	Pathogenic (known)	TOP
12	30	19	6.2 6.5	arr 17p12(14,099,564-15,482,833)x1	1.383	Unknown	Hereditary neuropathy with liability to pressure palsies (HNPP)	Pathogenic (unique)	Live birth
13	29	18^+3^	2.8 6.4	arr Xp22.33p11.1(168,551-58,526,888)x1 arr[hg19] Xp11.1q28(58,527,154-155,233,098)x3	58.358 96.706	De novo	46, X, i(Xq)^a^	Pathogenic (unique)	TOP
14	20	20^+6^	2.5 8.0	arr 22q11.21(18,648,855-21,269,224)x3	2.620	Unknown	22q11 duplication syndrome	Pathogenic (known)	TOP
15	28	20^+3^	4.1 5.5	arr 15q26.2q26.3(96,741,626-102,429,040)x1	5.687	De novo	16 MOM, 8 morbidity	Pathogenic (unique)	TOP
16	33	25^+5^	4.2 7.0	arr 7p14.3p14.1(30,131,466-37,881,701)x1	7.750	De novo	30 MOM, 12 morbidity	Pathogenic (unique)	TOP
17	35	20^+4^	6.1 5.3	arr 8p23.3p12(158,048-29,816,429)x3 arr 18q23(74,694,541-78,013,728)x1	29.658 3.319	Imbalance arising from a balanced parental rearrangement	8p23.1 deletion syndrome 10 MOM, 2 morbidity	Pathogenic (known)	TOP
18	27	19	Cystic hygroma 6.2			De novo	Trisomy 22 mosaicism	Pathogenic (unique)	TOP
19	36	20	3.5 6.0	arr 1p36.32p36.22(3,535,911-12,605,326)x1 arr 20q13.31q13.32(55,118,682-57,031,915)x1	9.069 1.913	De novo	1p36 microdeletion syndrome 11 MOM, 2 morbidity	Pathogenic (known)	TOP
20	31	20	3.0 5.4	arr 1q21.1q21.2(145,895,746-147,830,830)x1 arr 22q11.21(18,648,855-21,800,471)x1	1.935 3.152	Paternally inherited de novo	1q21.1 recurrent microdeletion 22q11 deletion syndrome	Pathogenic (known)	TOP
21	28	12^+4^	6.5 5.5	arr 9p24.3q13(208,454-68,317,844)x4	68.109	Unknown	Tetrasomy 9p	Pathogenic (unique)	TOP
22	28	21	3.4 6.5	arr 3p13p12.3(72,095,812-74,590,486)x1	2.4	De novo	7 OMIM	VOUS	Live birth

AF: amniotic fluid; NT: nuchal translucency; TOP: termination of pregnancy; VOUS: variants of uncertain significance; CRL: crown-rump length. ^a^Further confirmed by karyotype analysis.

**Table 4:** Comparison of sample size, analytical methods, and pathogenic findings in the present study with those in the published series.

Study	Number of patients	Cutoff of NT (mm)	Prior testing	CMA platform	Pathogenic CNVs, *n*(%)
Lund et al. [17]	94	3.5	QF-PCR	CGH (180K, Agilent)	12 (12.8)
Egloff et al. [16]	720	3.5	MLPA, BoBs, QF-PCR	CGH (60K, 180K, PrecytoNEM®, Agilent)	16 (2.7)
Pan et al. [21]	122	3.5	QF-PCR	SNP (250K, Affymetrix)	7 (5.7)
Scott et al. [22]	41	3.5	Karyotyping	CGH (60K, Agilent)	1 (2.4)
Schou et al. [15]	100	3.5	Karyotyping	CGH (BAC 3 Mb, targeted)	0 (0)
Huang et al. [23]	215	3.5	Karyotyping	CGH (44K, targeted)	0 (0)
Present study	499	2.5	QF-PCR	SNP (750K, Affymetrix)	21 (4.8)

BAC, bacterial artificial chromosome; BoBs, BACs-on-Beads; CGH, comparative genomic hybridization; CMA, chromosomal microarray analysis; CNVs, copy number variants; MLPA, multiplex ligation-dependent probe amplification; NT, nuchal translucency; QF-PCR, quantitative fluorescent polymerase chain reaction; SNP, single-nucleotide polymorphism.

## Data Availability

The data used to support the findings of this study are included within the article.

## References

[1] Nicolaides K. H., Azar G., Snijders R. J. M., Gosden C. M. (1992). Fetal nuchal oedema: associated malformations and chromosomal defects. *Fetal Diagnosis and Therapy*.

[2] Nicolaides K. H., Snijders R. J. M., Campbell S., Gosden C. M., Berry C. (1992). Ultrasonographically detectable markers of fetal chromosomal abnormalities. *The Lancet*.

[3] Snijders R., Noble P., Sebire N., Souka A., Nicolaides K. H. (1998). UK multicentre project on assessment of risk of trisomy 21 by maternal age and fetal nuchal-translucency thickness at 10–14 weeks of gestation. *The Lancet*.

[4] Senat M. V., De Keersmaecker B., Audibert F., Montcharmont G., Frydman R., Ville Y. (2002). Pregnancy outcome in fetuses with increased nuchal translucency and normal karyotype. *Prenatal Diagnosis*.

[5] Souka A. P., von Kaisenberg C. S., Hyett J. A., Sonek J. D., Nicolaides K. H. (2005). Increased nuchal translucency with normal karyotype. *American Journal of Obstetrics and Gynecology*.

[6] Baer R. J., Norton M. E., Shaw G. M. (2014). Risk of selected structural abnormalities in infants after increased nuchal translucency measurement. *American Journal of Obstetrics and Gynecology*.

[7] Ali M. M., Chasen S. T., Norton M. E. (2017). Testing for Noonan syndrome after increased nuchal translucency. *Prenatal Diagnosis*.

[8] Burger N. B., Bekker M. N., de Groot C. J. M., Christoffels V. M., Haak M. C. (2015). Why increased nuchal translucency is associated with congenital heart disease: a systematic review on genetic mechanisms. *Prenatal Diagnosis*.

[9] Spaggiari E., Stirnemann J., Ville Y. (2012). Outcome in fetuses with isolated congenital diaphragmatic hernia with increased nuchal translucency thickness in first trimester. *Prenatal Diagnosis*.

[10] Timmerman E., Pajkrt E., Maas S. M., Bilardo C. M. (2010). Enlarged nuchal translucency in chromosomally normal fetuses: strong association with orofacial clefts. *Ultrasound in Obstetrics and Gynecology*.

[11] de Mooij Y. M., van den Akker N. M. S., Bekker M. N., Bartelings M. M., van Vugt J. M. G., Gittenberger-de Groot A. C. (2011). Aberrant lymphatic development in euploid fetuses with increased nuchal translucency including Noonan syndrome. *Prenatal Diagnosis*.

[12] Fincham J., Pandya P. P., Yuksel B., Loong Y. M., Shah J. (2002). Increased first-trimester nuchal translucency as a prenatal manifestation of salt-wasting congenital adrenal hyperplasia. *Ultrasound in Obstetrics and Gynecology*.

[13] Levy B., Wapner R. (2018). Prenatal diagnosis by chromosomal microarray analysis. *Fertility and Sterility*.

[14] Wapner R. J., Martin C. L., Levy B. (2012). Chromosomal microarray versus karyotyping for prenatal diagnosis. *New England Journal of Medicine*.

[15] Schou K. V., Kirchhoff M., Nygaard U., Jørgensen C., Sundberg K. (2009). Increased nuchal translucency with normal karyotype: a follow-up study of 100 cases supplemented with CGH and MLPA analyses. *Ultrasound in Obstetrics and Gynecology*.

[16] Egloff M., Herve B., Quibel T. (2017). The diagnostic yield of chromosomal microarray analysis in fetuses with increased nuchal translucency: a French multicentre retrospective study. *Ultrasound in Obstetrics and Gynecology*.

[17] Lund I. C. B., Christensen R., Petersen O. B., Vogel I., Vestergaard E. M. (2015). Chromosomal microarray in fetuses with increased nuchal translucency. *Ultrasound in Obstetrics and Gynecology*.

[18] Lautrup C. K., Kjaergaard S., Brøndum-Nielsen K. (2008). Testing for 22q11 microdeletion in 146 fetuses with nuchal translucency above the 99th percentile and a normal karyotype. *Acta Obstetricia et Gynecologica Scandinavica*.

[19] Pergament E., Alamillo C., Sak K., Fiddler M. (2011). Genetic assessment following increased nuchal translucency and normal karyotype. *Prenatal Diagnosis*.

[20] Kearney H. M., Thorland E. C., Brown K. K., Quintero-Rivera F., South S. T. (2011). American College of Medical Genetics standards and guidelines for interpretation and reporting of postnatal constitutional copy number variants. *Genetics in Medicine*.

[21] Pan M., Han J., Zhen L. (2016). Prenatal diagnosis of fetuses with increased nuchal translucency using an approach based on quantitative fluorescent polymerase chain reaction and genomic microarray. *European Journal of Obstetrics and Gynecology and Reproductive Biology*.

[22] Scott F., Murphy K., Carey L. (2013). Prenatal diagnosis using combined quantitative fluorescent polymerase chain reaction and array comparative genomic hybridization analysis as a first-line test: results from over 1000 consecutive cases. *Ultrasound in Obstetrics and Gynecology*.

[23] Huang J., Poon L. C., Akolekar R., Choy K. W., Leung T. Y., Nicolaides K. H. (2014). Is high fetal nuchal translucency associated with submicroscopic chromosomal abnormalities on array CGH?. *Ultrasound in Obstetrics and Gynecology*.

[24] Grande M., Jansen F. A. R., Blumenfeld Y. J. (2015). Genomic microarray in fetuses with increased nuchal translucency and normal karyotype: a systematic review and meta-analysis. *Ultrasound in Obstetrics and Gynecology*.

[25] Kagan K. O., Avgidou K., Molina F. S., Gajewska K., Nicolaides K. H. (2006). Relation between increased fetal nuchal translucency thickness and chromosomal defects. *Obstetrics and Gynecology*.

[26] Kharrat R., Yamamoto M., Roume J. (2006). Karyotype and outcome of fetuses diagnosed with cystic hygroma in the first trimester in relation to nuchal translucency thickness. *Prenatal Diagnosis*.

[27] Maya I., Yacobson S., Kahana S. (2017). Cut-off value of nuchal translucency as indication for chromosomal microarray analysis. *Ultrasound in Obstetrics and Gynecology*.

[28] Hanson E., Bernier R., Porche K. (2015). The cognitive and behavioral phenotype of the 16p11.2 deletion in a clinically ascertained population. *Biological Psychiatry*.

[29] Ullmann R., Turner G., Kirchhoff M. (2007). Array CGH identifies reciprocal 16p13.1 duplications and deletions that predispose to autism and/or mental retardation. *Human Mutation*.

[30] Hannes F. D., Sharp A. J., Mefford H. C. (2009). Recurrent reciprocal deletions and duplications of 16p13.11: the deletion is a risk factor for MR/MCA while the duplication may be a rare benign variant. *Journal of Medical Genetics*.

[31] Mefford H. C., Cooper G. M., Zerr T. (2009). A method for rapid, targeted CNV genotyping identifies rare variants associated with neurocognitive disease. *Genome Research*.

